# Cough response to isocapnic hyperpnoea of dry air and hypertonic saline are interrelated

**DOI:** 10.1186/1745-9974-7-8

**Published:** 2011-10-14

**Authors:** Minna Purokivi, Heikki Koskela, John D Brannan, Kirsi Kontra

**Affiliations:** 1 Department of Respiratory Medicine, Kuopio University Hospital, P.O. Box 1777, 70211 Kuopio, Finland; 2 Department of Respiratory and Sleep Medicine, Royal Prince Alfred Hospital, Camperdown, NSW 2050, Australia; 3 Department of Pharmacy, Kuopio University Hospital, Kuopio, Finland

## Abstract

**Background:**

Mechanisms behind asthmatic cough are largely unknown. It is known that hyperosmolar challenges provoke cough in asthmatic but not in the healthy subjects. It has been postulated that isocapnic hyperpnea of dry air (IHDA) and hypertonic aerosols act via similar mechanisms in asthma to cause bronchoconstriction. We investigated whether there is an association between cough response induced by IHDA and hypertonic saline (HS) challenges.

**Methods:**

Thirty-six asthmatic and 14 healthy subjects inhaled HS solutions with increasing osmolalities administered via ultrasonic nebuliser until 15 cumulative coughs were recorded. The IHDA consisted of three three-minute ventilation steps: 30%, 60% and 100% of maximal voluntary ventilation with an end-point of 30 cumulative coughs. The challenges were performed on separate days at least 48 hours between them and within one week. Inhaled salbutamol (400 mcg) was administered before the challenges to prevent bronchoconstriction. The cough response was expressed as the cough-to-dose ratio (CDR) which is the total number of coughs divided by the maximal osmolality inhaled or the maximal ventilation achieved.

**Results:**

Cough response to IHDA correlated with the HS challenge (Rs = 0.59, p < 0.001). Cough response to IHDA was at its strongest during the first minute after the challenge. IHDA induced more cough among asthmatic than healthy subjects CDR being (mean ± SD) 0.464 ± 0.514 and 0.011 ± 0.024 coughs/MVV%, p < 0.001, respectively. Salbutamol effectively prevented bronchoconstriction to both challenges.

**Conclusions:**

Asthmatic patients are hypersensitive to the cough-provoking effect of hyperpnoea, as they are to hypertonicity. Cough response induced by IHDA and HS correlated well suggesting similar mechanisms behind the responses.

## Introduction

Chronic cough is a common diagnostic and therapeutic problem having prevalence up to 40% in population [1]. Cough can cause deterioration in the quality of life [2] and its economic burden is significant [3]. It also is the most common symptom of asthma [4]. Current therapies of asthma show little clinical efficacy on cough, and the treatment focus is on the underlying pathophysiology of disease. A recent consensus statement by the European Respiratory Society has highlighted the importance to further understand the mechanisms of cough through the development of valid tests to study cough and to identify and assess novel therapies to treat it [5].

It has been demonstrated that asthmatic cough can be independent of bronchial obstruction. For example, hypertonic saline provokes cough in asthmatic subjects who are pre-treated with inhaled salbutamol that is capable to block bronchoconstriction [6]. Unlike traditional cough provocation tests by capsaicin or citric acid [7-10], hypertonic cough provocation tests can differentiate asthmatic and healthy subjects [6]. Therefore, hypertonic challenge-provoked cough could be used in both diagnosing and evaluating treatment response of asthma on cough [11-13]. These findings also underline the potential clinical relevance of hypertonic saline (HS) challenge in investigating cough and in assessing cough therapies. A pathological function of sensorineural apparatus may be behind both asthmatic and chronic cough [6]. However, the precise mechanism of this cough is still unclear.

Isocapnic hyperpnoea of dry air (IHDA) challenge is thought to cause airway narrowing similarly to exercise by causing airway drying and leading to an increase in the osmolarity in the airway lining fluid [14-16]. Therefore, hyperpnoea of dry air can be regarded as a physiological stimulus. The inhalation of HS has been postulated to cause bronchoconstriction via the same mechanisms as exercise testing or hyperpnoea of dry air [16]. In addition, HS induced increase in osmolarity of the airway lining fluid is known to be a potent stimulator of airway sensory nerves and thus also cough [3]. Thus, we hypothesise that the cough responses to hypertonicity and hyperpnoea share similar mechanisms. To further investigate this subject, we compared the cough sensitivity to HS and IHDA in asthmatic and healthy subjects after pre-treatment with an inhaled beta2 agonist.

## Materials and methods

### Subjects

Thirty-eight subjects with asthma were recruited and entered the study from Kuopio University Hospital outpatient clinic. All asthmatic subjects were originally referred to this tertiary referral centre due to diagnostic uncertainty at primary care. The diagnosis of asthma was based on patient's history and clinical examination suggestive of asthma, together with objective evidence of reversible airway obstruction in spirometry or in ambulatory peak expiratory flow (PEF) measurements according to the GINA guidelines [17]. Fourteen healthy controls were recruited from the personnel of Kuopio University Hospital. The healthy subjects had no respiratory symptoms; however, atopy and history of smoking were not exclusion criteria. The exclusion criteria for all the subjects were febrile respiratory tract infection within six weeks, and post-salbutamol FEV_1 _less than 60% [18]. In addition, subjects with excessive spontaneous cough (>10 coughs in response to 0.9% saline inhalation) and subjects with fall of FEV_1 _more than 10% during neither of inhalation challenge used, were excluded from the study [19,20]. Thirty-six asthmatic and fourteen healthy subjects completed the study. The Research Ethics Committee, Hospital District of Northern Savo, Finland approved this study (31.10.2008 117//2008) and all subjects provided their informed consent for participation in the study. Subjects' characteristics are showed in table 1.

**Table 1:** Characteristics of the subjects.

	Asthma	Healthy control	p
n	36	14	
Gender (male/female)	10/26	2/12	
Age	40 (18-68)	37 (21-67)	
Atopy^#^	22	5	
Smoking (mean pack years)			
Current	9 (6)	0	
Previous	3 (11)	0	
Use of inhaled corticosteroids (ICS)	24	0	
Daily dose of ICS	541 ± 391 μg	-	
ASA intolerance	3	0	
Use of ACE inhibitors	5	0	
Exhaled nitric oxide	18.4 ± 15.0	14.6 ± 6.59	0.376
FEV_1 _(% from predicted)^¶^	90.0 (67-122)	92.8 (78-110)	0.418
Mean daily PEF variability (%)	7.90 ± 5.75	-	
Needed rescue medication doses during pre-test week	0.83 (0-4)	-	
CDR Hypertonic saline coughs/(mOsm/kg)	0.012 ± 0.010	0	<0.001
CDR Hyperpnoea of dry air coughs/MVV%	0.464 ± 0.514	0.011 ± 0.024	<0.001

Age, FEV_1 _and needed rescue medication doses mean (range). ^# ^Atopy was assessed as ≥3 mm mean wheal diameter in the skin prick test to at least one common aeroallergen. Daily dose of ICS, exhaled NO, mean daily PEF variability and CDR (cough-to-dose ratio) mean ± SD. ^¶ ^Reference values are those of Viljanen et al. [34]

### Non-permitted medication

The asthmatic patients were allowed to use their inhaled corticosteroids and long-acting β-2 agonists throughout the study but asked not to take them on the challenge days. All cough relieving drugs, leukotriene antagonists, and antihistamines were stopped at least three days before the first challenge. Short-acting β-2 agonists were withdrawn for at least 8 hours before the challenges.

### Protocol

All asthmatic subjects were observed over a one-week run-in period, during which they recorded peak expiratory flow (PEF) twice-daily using bronchodilator medication only when needed. Inhalation challenge with HS [6] and IHDA [21] were performed on separate days with at least 48 hours between the challenges within one week, during the same time of the day. Skin prick tests for common aeroallergens (Soluprick SQ^®^; ALK-Abello, Hörsholm, Denmark) and measurement of exhaled nitric oxide (eNO) (Sievers Model 280 NOA; Sievers Instruments Inc., Boulder, CO, USA) were performed to all subjects at the first visit.

### Hypertonic saline challenge

An ultrasonic nebuliser (De Vilbiss Ultraneb 3000, Sunrise Medical Ltd, Leicester, UK) with a one-way valve (Douglas Bag One-Way Air Valve, Harvard Apparatus, Holliston, MA, USA) was used to deliver the saline solutions. They were made by adjusting the sodium chloride (Natrii Chloridium Ph. Eur., Tamro Ltd, Helsinki, Finland) content of standard phosphate-buffered saline as described in detail previously [6]. In the beginning of the challenge, spirometry was performed three times (Model M9449, Medikro Ltd, Kuopio, Finland) and the largest FEV_1 _was recorded. Then the subject inhaled four inhalations of 100 mcg salbutamol (Ventoline Evohaler, GlaxoSmithKline Ltd, Stockley Park West, Uxbridge, Middlesex, UK) using a Volumatic chamber. The spirometry was repeated 15 minutes later. Thereafter the subject inhaled isotonic phosphate-buffered saline for 2 minutes using tidal breathing, and wearing a nose clip. The coughs occurring during the inhalation and two minutes after it were manually recorded by the investigation nurse as described previously [22]. Subsequently, the subject similarly inhaled solutions with osmolalities of 600, 900, 1200, 1500, 1800, and 2100 mOsm/kg. Oxygen saturation (SaO_2_) was monitored by pulse oximetry before and after salbutamol, and after every saline inhalation. The challenge was stopped if the subject asked for it, 15 or more cumulative coughs had been recorded [6], or the final solution was administered. Finally, the spirometry was repeated.

### Isocapnic hyperpnoea of dry air challenge

The challenge was performed as described earlier by Rodwell et al. [21] with some modifications. In brief, the subject inhaled dry compressed air (Woikoski, Varkaus, Finland) containing 5% CO_2 _which prevents alkalosis during the challenge. The apparatus used had a target balloon (Direct Fillsingle Bag Set, Harvard Apparatus Ltd, Edenbridge, Kent, UK) in the inspiratory line, between the rotameter (Rotameter, Aalborg Instruments, Kytölä, Muurame, Finland) and the mouthpiece (Douglas Bag One-Way Air Valve and Mouthpiece, Harvard Apparatus Ltd, Edenbridge, Kent, UK). A demand valve (Aga Gas Ltd, Lidingö, Sverige) was utilised in the setting of the gas flow. The subject breathed gas from the target balloon. The subject was encouraged to keep the size of the target balloon constant with increasing breathing frequency and volume when the airflow to balloon increased during the different steps of the challenge. The challenge began with measurement of baseline FEV_1_. Then the subject inhaled four inhalations of 100 mcg salbutamol (Ventoline Evohaler, GlaxoSmithKline Ltd, Stockley Park West, Uxbridge, Middlesex, UK) using a Volumatic chamber, and the measurement of FEV1 was repeated after 15 minutes. The best post-salbutamol FEV_1 _was used to calculate the maximal voluntary ventilation (MVV), taken as 35 × post-salbutamol FEV_1_. The challenge commenced breathing at 30% MVV for three minutes. Coughs were counted manually by the investigation nurse during the challenge and up to 10 minutes after it [22]. Then two FEV_1 _measurements were performed. The challenge continued with ventilation at 60% MVV for three minutes, and succeeded with cough counting and two FEV_1 _measurements. Finally the subject ventilated at 100% MVV for three minutes, and cough counting and two FEV_1 _measurements were repeated. SaO_2 _was monitored by pulse oximetry in the beginning of the challenge and after each step. The challenge was stopped if the subject asked for it, 30 or more cumulative coughs were recorded, or the fall in FEV_1 _was 10% or more compared to the post-salbutamol value.

### Statistical analysis

Saphiro-Wilkins test was used for normality testing due to the sample size (n < 50). Correlation between challenge induced cough responses was determined with Spearman's rank correlation coefficient (Rs). Cough response, the number of coughs as a product of the quantity of the stimulus, was expressed as coughs-to-dose ratio (CDR). In HS challenge CDR was calculated as cumulative coughs/final osmolality. In IHDA challenge CDR was determined as cumulative coughs/final MVV%. Differences in CDR values between groups were assessed with Mann-Whitney U-test. Normally distributed values were compared with Student's t-test and paired samples t-test. Values are expressed as mean and standard deviation. A p-value of <0.05 was considered to be statistically significant. All analyses were carried out using SPSS for Windows 15.0 (SPSS Inc.™, Chicago, USA).

## Results

**Figure 1:**
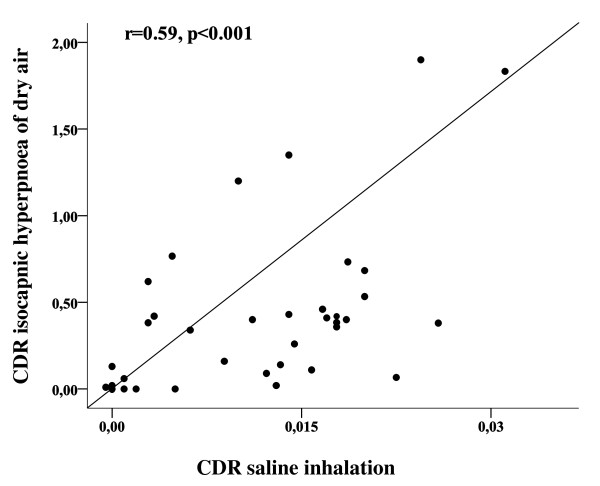
**Coughs-to-dose ratio (CDR) of isocapnic hyperpnea of dry air challenge in relation to CDR of hypertonic saline inhalation challenge of asthmatic subjects (n = 30)**.

The cough response to IHDA correlated well to the cough response of hypertonic aerosol among asthmatic subjects with a relationship between coughs-to-dose ratios Rs = 0.59, p < 0.001 (Figure 1.). In addition, the relationship was maintained when comparing the cumulative coughs at the end of the saline and IHDA challenges with a Spearman correlation coefficient of 0.46, p = 0.01.

**Figure 2:**
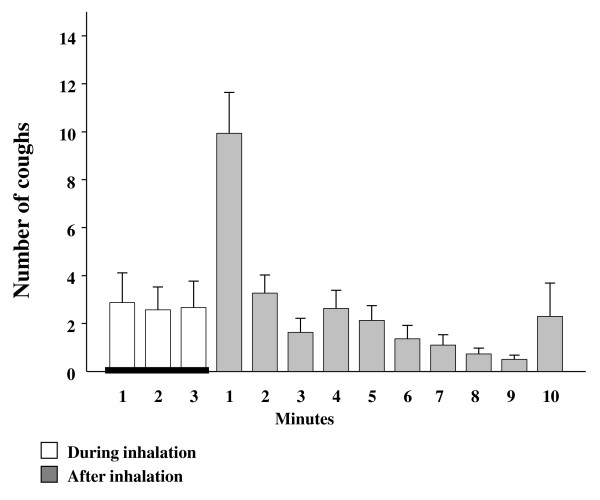
**Number of provoked coughs (means and standard errors) at each stage of all isocapnic hyperpnea of dry air challenges of asthmatic subjects (n = 30)**.

**Figure 3:**
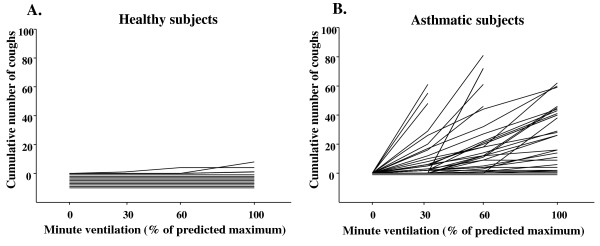
**The cumulative number of coughs in relation to minute ventilation evoked by isocapnic hyperpnea of dry air challenge in each (a) healthy (n = 10) and (b) asthmatic (n = 30) subject**. The horizontal lines at or below zero indicate subjects who did not cough at all.

Cough response to IHDA was at its strongest during the first minute of the post-hyperventilation period, then ceasing rapidly (Figure 2.). In some subjects cough was prolonged following the stimulus and cough occurred through-out the post hyperventilation period. IHDA and HS were more potent at inducing cough in asthmatics than in healthy subjects, p < 0.001 (Table 1). Further, the asthmatic subjects started to cough at lower minute ventilation than the healthy subjects (Figure 3). The cough indices separated the asthmatic and healthy subjects but eNO and FEV_1 _measurements did not (Table 1.).

Four subjects from the asthmatic group and one healthy subject failed to achieve their target ventilation MVV100% during the last step of IHDA. Despite this, three of the mentioned asthmatic subjects had cough reaction between 29-34 coughs when minimum target for cessation was 30 cumulative coughs, and the healthy subject did not cough at all.

There were no statistically significant differences in FEV_1 _between the two challenges in either study group. FEV_1 _(expressed as % from predicted) of asthmatic subjects was 89.0 ± 2.31 before the IHDA and 89.6 ± 2.25 before the HS, and 91.1 ± 2.22 and 92.8 ± 2.55 of the healthy subjects, respectively. Accordingly, FEV_1 _after IHDA and HS was 92.4 ± 2.08 and 92.7 ±2.13 among asthmatic, and 96.7 ± 2.28 and 97.6 ± 2.69 among healthy subjects. Post-salbutamol FEV_1 _did not differ significantly from the FEV_1 _at the end of challenge in either of the tests nor in either of the groups: Salbutamol prevented a fall of FEV_1 _among asthmatic subjects with the reductions in FEV_1 _only a mean (range) 0.32% (-3.8% - +5.1%) after HS and -0.9% (-6.7% - +9.3%) after IHDA. There was no clinically significant reduction in SaO_2 _in association to hyperpnoea with the saturation being 97.4 ± 0.2% before and 97.1 ± 0.2% after the challenge. The same was observed for HS with the mean SaO_2 _before and after the challenge being 96.9 ± 0.2% and 97.1 ± 0.3%. However, two asthmatic subjects had decrease >4% in SaO_2 _during coughing in the end of HS challenge and this was not related to any change in FEV_1_.

## Discussion

The results of this study show that asthma patients with cough response to HS also have similar cough response to IHDA. In addition, the profile of the cough response following IHDA is similar to that of HS in our previous study [6]. The IHDA induced cough is at its strongest during the first minute after the challenge followed by rapid decline in the response. These findings support our hypothesis that the mechanism behind the cough response is the same in these challenges.

It has been proposed previously that IHDA causes water loss from the airways similarly to exercise challenge [14]. This leads to changes in osmolarity of the airway lining fluid and probably also in the epithelial cell. It has been further hypothesised that this is the main mechanism behind the bronchial obstruction in hyperpnoea- and exercise-induced asthma [23]. Several reports support this hypothesis. The results of Smith et al. have demonstrated that bronchial obstruction provoked by inhalation of 4.5% saline correlated to that of IHDA in both intensity and onset of action [15]. The similarity of the airway responses to exercise and hyperpnoea of dry air [16], and inhalation of mannitol [24] further support the concept that these indirect challenges initiate same pathophysiological responses in the airways to cause bronchoconstriction.

In this study, in addition to the cough response correlating strongly between HS and IHDA challenges, we found that the cough responsiveness was at its highest during the first minute following the IHDA, mimicking previous findings after inhalation of HS [6]. The peak of the cough response appeared earlier than the largest decrease of FEV_1 _reported in the earlier studies after either IHDA or HS challenge where the maximum airway response can be expected five minutes after the withdrawal of the stimulus [15,25]. These findings together suggest that mechanism behind the evoked cough response after HS and IHDA challenges is likely to be similar. In the contrast to the bronchoconstrictive response to hypertonicity and hyperpnoea, the cough response to these stimuli is probably not associated with mast cell derived mediator release [26]. The putative role of mast cell in the bronchoconstrictive response to hypertonicity and hyperpnoea is highlighted by the strong inhibitory effects of mast-cell stabilising agents on this response [11,27,28]. However, the present and our previous studies demonstrate that these drugs, i.e. salbutamol and nedocromil are without effect on the cough response on these stimuli [6,11].

The appearance of the cough response to IHDA and hypertonicity with delay, i.e., after the challenges, has previously been considered as a proof for an indirect stimulation of the cough receptors via a mediator release [6]. However, Lavorini et al. have recently reported that both exercise and isocapnic hyperpnoea of dry air can down regulate the sensitivity of the cough reflex [29]. This finding is important and may actually explain the delayed cough response to IHDA and hypertonicity shown in the present and our previous study [6].

Previous studies utilising capsaicin in cough induction have not been able to show any clinical use of it in diagnosing asthma or in evaluating treatment response of asthmatic cough [8,9,30,31]. In addition, capsaicin challenge does not seem to have any correlation to mannitol in the assessment of chronic cough [32]. Capsaicin as well as citric and tartaric acids are known to activate airway sensory nerves via the stimulation of the type 1 vanilloid receptor (TRPV 1). In contrast, HS, which is a robust activator of airway sensory nerves, acts independently of TRPV1 [3]. The present results suggest that IHDA may act via the same mechanism as HS in cough induction. Mannitol, another hyperosmolar stimulus, also may utilise the same pathway [33]. However, the recognition of the precise sensory neural mechanism responsible for the HS and IHDA induced cough is needed.

This paper presents a new type of physiological cough provocation test, IHDA after salbutamol pre-treatment. One could criticise the use of different end-points in the challenges. The end-point of fifteen coughs for saline challenge had been previously determined [6]. However, the end-point of thirty coughs for IHDA challenge was an estimation. Determination of an end point requires a repeatability study. So far, repeatability study of IHDA as an cough challenge has not been performed. In future, an accurate end-point should be evaluated also for IHDA induced cough. There were no clinically significant decreases in FEV_1 _or oxygen saturation levels during either of the challenges utilised in this study. At the present, both HS and IHDA seem to be physiological and safe challenges for cough research. Though challenge induced cough may be uncomfortable to the patient, it is safe to perform as no adverse events have been observed.

In conclusion, cough response to hypertonic saline compares well to the cough sensitivity to the real life cough stimulant, hyperpnoea of dry air. This report suggests that safety and practicality of HS and IHDA induced cough response could be utilised in investigating mechanisms of cough.

## Abbreviations

Following abbreviations are used in this manuscript: CDR: coughs to dose ratio; eNO: exhaled nitric oxide; FEV_1_: forced expiratory volume in one second; HS: hypertonic saline; IHDA: isocapnic hyperpnoea of dry air; MVV: maximal voluntary ventilation; PEF: peak expiratory flow; SaO_2_: oxygen saturation.

## Competing interests

The authors declare that they have no competing interests.

## Authors' contributions

M. P. took part in study planning, recruited the subjects, and collected and analysed the data. She had the main responsibility of writing of the manuscript. H. K. took part in study planning, recruited the subjects, and took part in writing process. J. B. took part in study planning and writing process.

K. K. took part in study planning, was responsible for production of hypertonic saline solutions and quality control, and took part in writing process. All authors have read and approved the final manuscript.
